# MammaPrint Feasibility in a Large Tertiary Urban Medical Center: An Initial Experience

**DOI:** 10.6064/2012/942507

**Published:** 2012-12-31

**Authors:** C. Francisco Espinel, Shaughn Keating, Hanina Hibshoosh, Bret Taback, Kathie-Ann Joseph, Mahmoud El-Tamer, Sheldon Feldman

**Affiliations:** ^1^Division of Breast Surgery, Department of Surgery, Columbia University Comprehensive Breast Center, New York, NY 10032, USA; ^2^Columbia University College of Physicians and Surgeons, New York, NY, USA

## Abstract

*Background*. The MammaPrint (MP) diagnostic assay stratifies breast cancer patients into high- and low-risk groups using mRNA analysis of a 70-gene profile. The assay is validated for assessment of patients with estrogen receptor positive or negative tumors less than 5 cm with 3 or fewer malignant lymph nodes. TargetPrint (TP) is an assay for assessing estrogen, progesterone, and HER2-neu receptor status based on mRNA expression. A potential limitation of these assays is that they require an evaluation of fresh tissue samples. There is limited published experience describing MP or TP implementation. *Methods*. Over 10 months, 4 breast surgeons obtained samples from 54 patients for MP/TP analysis. The samples were analyzed by Agendia Labs. The tumors were independently evaluated for receptor status using immunohistochemistry (IHC). Retrospectively, we identified patients who were assessed by MP/TP during this period. Patients who underwent OncotypeDx evaluation were also identified. *Results*. Of the 54 patients receiving MP, 4 were found ineligible for MP risk assessment because >3 lymph nodes were found to be malignant. Out of all eligible patients, 14/50 (28%) had samples whose quantity of tumor was not sufficient for analysis (QNS). Out of eligible patients with tumors <1 cm, 7/8 (88%) had QNS samples. 7/42 with tumors ≥1 cm (17%) had QNS samples. Nine patients had discordant receptor results when evaluated by IHC versus. TP. Of patients who also underwent OncotypeDx testing, 6/14 (43%) had discordant results with MP. *Conclusions*. This study indicates that using MP/TP assay is feasible in a tertiary care center but there may be utility in limiting MP testing to patients with tumors between 1 and 5 cm due to high likelihood of uninformative results in subcentimeter tumors. Further study is needed to explore the discordance between oncotype and MP results.

## 1. Background

 The MammaPrint (MP) 70-gene expression profile was developed to help predict clinical outcome for breast cancer patients and has been validated in prior studies in both estrogen receptor (ER) positive and negative breast cancers [1–5]. MP estimates risk for distant breast cancer metastases by measuring the mRNA expression levels of a panel of 70 genes via microarray and then applying an algorithm that classifies the tumors dichotomously as either high or low risk [1]. High risk is equivalent to a >29% chance for distant metastasis at ten years without adjuvant therapy, and low risk is equivalent to a <10% chance for distant metastasis without adjuvant therapy [3]. Although the initial development of the gene panel and risk algorithm was designed using a cohort of breast cancer patients with negative lymph nodes, subsequent European studies have validated MP's prognostic value in patients with up to 3 tumor positive lymph nodes [2, 5].

The original cohort consisted only of patients younger than 55 years of age, and the 3 major European validations of the MP test subsequently capped the age of diagnosis at 53, 61, and 71 [2, 3, 5]. An additional analysis on 100 frozen tumor samples from Massachusetts General Hospital, in which the majority of patients were greater than the age of 55, identified a significant difference in the positive predictive value for metastasis-free survival of MP from the Dutch validation study (12% versus 52%), suggesting that biological differences in the tumors from older patients may lead to differences in the clinical utility of MammaPrint testing [4]. Moreover, none of the validations have reported MP results by race, although it has been shown that different ethnicities may have genetically different tumors [6]. Therefore, the prognostic utility of MP may differ in postmenopausal, ethnically diverse American urban patient populations from the European patient populations in which it was developed and initially validated.

The risk assessment produced by MP has demonstrated significant variation from “traditional” risk assessment guidelines based on clinical parameters (e.g., St. Gallen, NIH criteria, and Adjuvant Online) and has been consistently demonstrated to be more accurate [2, 3, 7]. The high negative predictive value of MP for metastasisfree survival suggests that treatment plans based on MP rather than conventional criteria could potentially identify a population of biologically favorable tumors in which adjuvant chemotherapy could be withheld [8]. A prospective multicenter European trial, MINDACT (Microarray in Node Negative Disease May Avoid Chemotherapy), is currently underway to further validate this hypothesis by taking patients with discordant MP and traditional risk assessments and randomizing them to receive adjuvant chemotherapy based on either conventional or MP results when those results are discordant [8, 9].

MP analysis is RNA microarray based that requires fresh tissue samples [1]. Logistically, this presents a potential challenge because the surgical specimen must be transported fresh to pathology for inking and obtaining an adequate sample for analysis in less than one hour from resection. This requires preplanning and coordination with the pathology department. This logistical challenge has been identified as a disadvantage when compared to the predictive 21-gene assay OncotypeDx, which relies on a real-time polymerase chain reaction (RT-PCR) that can be performed on formalin-fixed paraffin-embedded (FFPE) sections [10]. However, MP is validated for use in both ER positive and negative breast cancers, as compared to OncotypeDx, which is validated for use in only ER positive cancers. Therefore, MP has the potential to obtain prognostic information for more breast cancer patients, given its application to hormone receptor positive and negative tumors. However, in contrast to OncotypeDx, MP is not currently validated for use in predicting response to treatment and thus OncotypeDx has more clinical utility in situations where an ER positive patient is eligible for either oncotype or MP testing [10]. 

In conjunction with MP analysis, the TargetPrint (TP) array determines estrogen, progesterone (PR), and HER2-neu (HER2) receptor status to a high degree of concordance to conventional immunohistochemical (IHC) or fluorescence in situ hybridization (FISH) analysis [11, 12]. To determine positive versus negative receptor expression, the ER, PR, and HER2 mRNA extracted from fresh tumors were quantified and a microarray threshold was determined that led to the highest concordance with IHC/FISH results [11]. In the original cohort used to set the optimal thresholds, the concordance was not 100% for any of the receptors, demonstrating an overlap in mRNA levels between tumors that are receptor positive by IHC/FISH and tumors that are receptor negative by IHC/FISH. The subsequent validations were preformed on separate cohorts of patients and compared to IHC/FISH results, and concordance rates of between 83 and 97% were observed [11, 12].

## 2. Methods

The patient population was from the Division of Breast Surgery at Columbia University Medical Center, with data collected from December 2008 to September 2009. Each patient who underwent MP testing over this interval was independently identified for MP eligibility by one of the four primary breast surgeons in the department. Generally, all patients with tumors greater than 1 cm in maximum diameter were considered eligible for testing. Tumor samples were collected during mastectomy or lumpectomy or core needle biopsy prior to tumor resection. The lumpectomy or mastectomy specimens were transported fresh from the operating room to the pathology lab. A dedicated technician facilitated the transport and specimen retrieval. At a minimum, a 3 mm specimen from the periphery of the tumor was obtained and placed in a RNA preserving solution for transport to Agendia Labs. The core needle biopsy specimens were processed in a similar fashion. The RNA was extracted and analyzed for MP and TP profile in Agendia's central laboratory in California, and the results were sent electronically to the attending breast surgeon. The tumors were independently evaluated by the Pathology Department at Columbia University Medical Center for histology and standard receptor status assessment. ER and PR status was determined by using IHC staining and HER2-neu status by IHC, or if HER2 was 2+, FISH was sent. 

An IRB approved database of patients undergoing MP and/or TP was established to analyze results. Data points included surgeon, age, ethnicity, specimen type (mastectomy, lumpectomy, or core biopsy), histological tumor type, number of tumor positive lymph nodes (if axillary lymph node dissection or sentinel lymph node biopsy was performed), tumor size, pathologic tumor stage, receptor status as determined by IHC and/or FISH, and OncotypeDx recurrence score (if available) along with MP and TP results.

Retrospectively, we identified all of the patients seen by the Department of Breast Surgery with ER positive or negative tumors, ≤3 tumor positive lymph nodes, and tumors less than 5 cm but greater than 1 cm in maximum diameter. A capture ratio was then calculated as all the patients meeting these criteria that were assessed for MP divided by “all the patients matching these criteria.”

## 3. Results

A total of 54 breast cancer patients had samples submitted for MP analysis and 51 of patients had concomitant TP analysis. Of these 54 patients, 7 had more than one MP and/or TP report returned on the submitted sample, secondary to inadequate amounts of assessable tumor in the initial analysis that required a second analysis of the sample. This resulted in a total of 62 MP reports and 59 TP reports received. The ethnic distribution of the 54 MP patients was 36 (67%) Caucasian, 9 (17%) Hispanic, 6 (11%) Black, and 3 (6%) Asian. The surgical specimens included 19 mastectomies, 32 lumpectomies, and 3 core biopsies. One patient receiving a core biopsy did not receive a subsequent evaluation of their lymph nodes. OncotypeDx was performed on 14 patients who underwent MP. Nine of these patients were determined to have low risk of recurrence (OncotypeDx recurrence score < 18) and 5 with intermediate risk (recurrence score 18–30). The risk results are tabulated in Table 1. Of the 54 unique patients, 4 had >3 positive lymph nodes and were therefore ineligible for MP risk assessment. For the remaining 50 unique samples, 29 (58%) were high risk, 7 (14%) were low risk, and 14 (28%) had an insufficient amount of tumor within the sample for analysis (QNS). Out of this group of 50 samples, 8 were <1 cm in maximum diameter and 1 (12%) of these was high risk, while the remaining 7 (88%) were QNS. Out of the seven low risk patients, three had one or more positive lymph nodes. 

Three patients did not undergo TP analysis, yielding a total of 59 TP assessments on 51 individual patients (Table 2). The TP results, including the final reports of those patients who received multiple results, identified 11 ER negative, 26 ER positive, 18 PR negative, 19 PR positive, 32 HER2-neu negative, 5 HER2-neu positive, and 14 samples QNS (quantity not sufficient) for TP receptor mRNA assessment. Ten discordant results were found in eight patients when comparing receptor status (2 ER, 6 PR and 2 Her2-neu) compared to IHC/FISH. This results in a ratio of overall (for combined ER/PR/Her2-neu) discordant outcome to independent, non-QNS receptor assessments of 10/111 (9%).

Month-by-month review showed that 17 unique patients received MP in the first 5 months of the study and 37 unique patients received MP in the last 5 months of the study, an increase of 117%. Review of the proportion of total unique samples sent for MP analysis by each of the primary surgeons from the department showed that surgeon A sent 26/54 (48%) of the total samples for MP, B sent 13/54 (24%), C sent 8/54 (15%), and D sent 5/54 (9%). Of the 54 unique MP patients, 12 did not match the eligibility criteria used in constructing our capture ratio. Exclusion of these patients was based on postsurgical histopathologic evaluation, which revealed 4 patients with more than 3 positive lymph nodes and 8 patients with tumors smaller than 1 cm in maximum diameter. 139 patients with ER positive or negative invasive cancers with maximum diameters ≥1 cm and ≤3 positive lymph nodes were retrospectively identified over the study interval, and a capture ratio of 42/139 (30%) was calculated. 

## 4. Discussion

The main goal of this study was to determine whether it was feasible to implement MammaPrint testing in a large, urban, tertiary care center. A notable finding in this regard was the high rate of QNS results; 14/50 (28%) of all results being returned QNS. The subgroup of patients with tumors from 1–5 cm, only 7/42 (17%), had QNS results. When evaluating small (<1 cm) tumors, pathologists may be concerned in giving up any diagnostic tissue. This may affect the pathologists' comfort in excising an adequate amount of tissue from smaller tumors, as they may face the choice between sending tumor tissue for MP analysis versus depositing it in the institutional tumor bank. Although further study is needed due to our small sample size, setting a minimum size for MP specimens may result in lower QNS rates in tertiary care centers, leading to more cost-effective utilization of MP and increasing its clinical utility. 

However, there would be significant ramifications if MP was restricted to patients with tumors >1 cm. Although OncotypeDx has the benefit of allowing tumor tissue selection after routine pathology processing, thus lowering concerns about sacrificing tumor tissue with small primary tumors, the test is not validated in ER negative tumors. Therefore, a gap in currently commercially available genomic profile options would exist for patients with subcentimeter ER negative tumors.

Out of the >1 cm tumors, 3 out of 4 lobular cancers had QNS results returned. Although our patient series was not large enough to draw any statistically significant conclusions about this pattern of QNS results, it may warrant further investigation of difficulties in gathering appropriately sized tumor samples from lobular cancers with high enough proportions of tumor cells for MP analysis. There were no trends in QNS results between mastectomy, lumpectomy, or core biopsy specimens, suggesting that technique used to collect the specimen is less important that the absolute size of the tumor. Notably, 3 out of 7 samples classified as low risk were from patients with one or more positive lymph nodes. This affirms MP's ability to provide risk assessments that are discordant from traditional assessments, which may lead to avoidance of unnecessary chemotherapy if the MINDACT trial proves successful.

 In addition to the quality of samples, the logistics of identifying MP eligible patients and collecting the individual samples is of utmost importance if MP is to be effectively utilized. Coordination is required with the pathology department, and a minimum amount of tumor tissue must be available to send for MP testing. In our experience, the logistical issues were shown to be resolvable. The capture ratio of 30% among patients with larger tumors shows that many patients in which MP testing might be informative did not undergo this testing. The question is whether this low capture rate is due to fundamental limitations in the logistics of fresh MP specimen collection or due to individual variation in the expertise of each surgeon in organizing specimen collection and his/her proclivity towards ordering this assessment. Breakdown of results by surgeon and by month suggests that much higher rates could have been achieved. The disparity between surgeons, with surgeon A collecting over 5 times as many MP specimens as surgeon D, shows it is feasible to increase the capture ratio without any change in the logistics of specimen collection. Similarly, the 117% increase in specimen collection in the second half of the study suggests that, as surgeons became more comfortable with the collection process, a much higher capture ratio was achieved. We are currently improving our standardization of case capture by using a check-off box on the operating room booking slip. Thus, logistically it appears feasible to collect fresh MP and TP samples in a large tertiary care center at high capture rates. 

 Generally speaking the MammaPrint results were of great interest to the medical oncologists making systemic therapy decisions. They were however reluctant to use the MP results independently and did not feel comfortable not recommending chemotherapy for node positive patients with low risk scores. It is unlikely that clinical practice will be greatly altered by MP until the MINDACT trial is completed. In the interim, subgroup selection for MP analysis suggests that patient who have tumors > 1 cm and are ER− can potential benefit; a low risk score could change systemic therapy recommendation.

The 9% discordance in TP receptor assessments to IHC/FISH is similar to the 8% discordance found in a similar study in a German population, highlighting that the diversity of our patient population did not affect the accuracy of TP assessment [12]. The discordant results confirm previous studies showing that mRNA levels of the receptor genes may not be perfectly correlated to protein receptor expression as determined by IHC [11, 12]. Which methodology is more predictive of response to therapy remains to be determined. TP assessment may act as a cost-effective adjunct when IHC/FISH results are inconclusive or suggest reevaluation of different samples when available of receptor expression studies.

Although the study did not involve enough OncotypeDx patients to draw statistically significant results, the 6 patients with discordant results, that is, high risk MP results and either low or intermediate risk OncotypeDx results, may represent fundamental differences between the two gene assays' risk results and suggest further investigation into disparities between MP and OncotypeDx is warranted. Although MP is used currently for its prognostic value, with the MINDACT trial nearing accrual, MP may soon become a useful predictive tool for planning treatment of breast cancer. Therefore, in ER positive patients who are eligible for both OncotypeDx and MP, discordant results would result in different treatment suggestions for the same individual. It will be of utmost importance to identify the nature and frequency of these discordancies through further study before MP comes into use as a predictive tool. We are continuing to collect MP and OncotypeDx data on prospective patients with tumors greater than 1 cm toward the goal of identifying all patients who may respond to endocrine or HER2 directed therapy. We also plan to further explore the utility of MP for invasive lobular carcinoma. Genomic evaluation of our ethnically diverse patient population may allow better understanding of variations among subgroups.

## Figures and Tables

**Table 1:** Summary of MammaPrint risk assessments.

	Total	MP high risk (%)	MP low risk (%)	QNS (%)
Total, unique	54	33 (61%)	7 (13%)	14 (26%)
>3 LNs positive	4	4 (100%)	0	0
MP indicated tumors*	50	29 (58%)	7 (14%)	14 (28%)
<1 cm max diameter	8	1 (12%)	0	7 (88%)
0 positive LNs^†^	34	19 (56%)	4 (12%)	11 (32%)
1–3 positive LNs^†^	15	9 (60%)	3 (20%)	3 (20%)
ER+, PR+, HER2+	2	2 (100%)	0	0
ER−, PR−, HER2+	4	3 (75%)	1 (25%)	0
ER+, PR+, HER2−	29	16 (55%)	4 (14%)	9 (31%)
ER−, PR−, HER2−	6	5 (83%)	0	1 (17%)
ILC	4	1 (25%)	0	3 (75%)
IDC	46	28 (61%)	7 (15%)	11 (24%)
Caucasian	35	22 (63%)	2 (6%)	11 (31%)
Non-Caucasian	15	7 (47%)	5 (33%)	3 (20%)
OncotypeDx low risk	9	3 (33%)	2 (22%)	4 (44%)
OncotypeDx intermediate risk	5	3 (60%)	0	2 (40%)

MP: MammaPrint; QNS: quantity not sufficient; LN: lymph node; ER: estrogen receptor status by IHC; PR: progesterone receptor status by IHC; HER2: HER2-neu receptor status by IHC/FISH; ILC: invasive lobular carcinoma; IDC: invasive ductal carcinoma.

*MP indicated in current international guidelines for stage 1 or 2, ER+ or − invasive breast cancer <5 cm in maximum diameter with ≤3 LNs tumor positive.

^†^One core biopsy specimen was not subsequently assessed for lymph node status.

**Table 2:** Summary of TargetPrint receptor results.

		TargetPrint results		
	Positive	Negative	QNS	Discordant
ER	26	11	14	2
PR	19	18	14	6
HER2	5	32	14	2

ER: estrogen receptor; PR: progesterone receptor; HER2: HER2-neu receptor; QNS: quantity not sufficient.
